# Depressive and Anxiety Symptoms at Initial Psychiatric Assessment in Patients Entering Hematopoietic Stem Cell Transplantation Protocols: A Cross-Sectional Study Using PHQ-9 and GAD-7

**DOI:** 10.7759/cureus.111561

**Published:** 2026-06-26

**Authors:** Alejandro Salazar-Rodriguez, Deldhy Nicolás Moya-Sánchez, Elia Francisca Espinoza-de-Ávila, Luara Luz Arana-Luna, Marta Georgina Ochoa-Madrigal, Litzy Vazquez-Reynoso

**Affiliations:** 1 Psychiatry and Liaison Psychiatry, National Medical Center "20 de Noviembre", Mexico City, MEX; 2 Hematology and Medical Oncology, National Medical Center "20 de Noviembre", Mexico City, MEX; 3 Psychiatry and Child Psychiatry, National Medical Center "20 de Noviembre", Mexico City, MEX; 4 Psychiatry, National Medical Center "20 de Noviembre", Mexico City, MEX

**Keywords:** anxiety, bone marrow transplantation, depression, gad-7, hematopoietic stem cell transplantation, liaison psychiatry, mexico, phq-9

## Abstract

Objective

Depression and anxiety are common across hematopoietic stem cell transplantation (HSCT) and predict poorer adherence and worse outcomes, yet their burden at the initial psychiatric evaluation has not been documented in Mexican patients. Brief validated screeners such as the Patient Health Questionnaire-9 (PHQ-9) and the Generalized Anxiety Disorder-7 (GAD-7) could detect these symptoms at this early, accessible window, before conditioning begins. We aimed to describe the frequency and severity of depressive and anxiety symptoms, assessed by the PHQ-9 and GAD-7, in patients entering an HSCT protocol at a Mexican tertiary center during their initial psychiatric evaluation.

Methods

A retrospective, cross-sectional, observational study was conducted at the Centro Medico Nacional "20 de Noviembre" (ISSSTE), Mexico City. We reviewed records of 104 adult patients evaluated by the Psychiatry Department as part of the HSCT protocol between January 2024 and November 2025; 103 met the inclusion criteria. PHQ-9 and GAD-7 scores, demographic data, hematological diagnosis, and transplantation type were extracted. Clinically significant depression was defined as PHQ-9 ≥10 and clinically significant anxiety as GAD-7 ≥10. Data were analyzed with descriptive statistics, non-parametric tests, and Fisher's exact tests (alpha=0.05).

Results

The median age was 51 years (interquartile range [IQR] 38.5-60.5); 55.3% were men. The most common diagnosis was multiple myeloma (48.5%); 66% were scheduled for autologous transplantation. Median PHQ-9 was 0 (IQR 0-2); clinically significant depression was detected in eight patients (7.8%; 95% confidence interval [CI] 4.0-14.6). Median GAD-7 was 0 (IQR 0-1.5); clinically significant anxiety was present in two patients (1.9%; 95% CI 0.5-6.8). Co-occurrence of both was also 1.9%. Patients aged 18-35 years had a fourfold higher prevalence of mild anxiety compared with those aged 36-55 years (prevalence ratio [PR]=4.2; 95% CI 1.17-15.10; p=0.026). PHQ-9 and GAD-7 scores correlated positively (p<0.0001).

Conclusions

At the initial psychiatric encounter, most HSCT patients reported minimal affective symptoms; the timing of assessment, before conditioning begins, and the prior selection of medically and psychosocially stable candidates likely explain these low rates. The 7.8% rate of clinically significant depression and the concentration of mild anxiety in younger patients support systematic screening with validated tools at this early stage. The age-anxiety association is confounded with diagnosis and transplantation type and requires replication in larger samples. Longitudinal studies are needed to characterize symptom trajectories throughout the transplantation process.

## Introduction

Bone marrow transplantation (BMT) - more precisely, hematopoietic stem cell transplantation - has substantially improved survival for patients with acute leukemias, lymphomas, myelodysplastic syndromes, and other severe hematological disorders [1]. The procedure is technically complex, but its psychological burden is equally demanding: prolonged hospitalization, enforced isolation, severe conditioning toxicities, immune compromise, and a sustained life threat combine to create one of the most emotionally challenging experiences in clinical medicine [2,3].

International data consistently document high rates of affective disturbance throughout the BMT trajectory. Depression occurs in 20-50% of patients depending on the assessment time point, instrument, and sample characteristics [4-7]. Anxiety, particularly generalized or anticipatory forms, affects up to one-third of hospitalized transplant recipients [7]. These symptoms are not merely distressing: they are associated with poorer treatment adherence, longer hospital stays, slower immunological recovery, and, in some series, higher mortality [8-12].

Screening tools validated for medical populations provide an efficient means to detect these problems early. The Patient Health Questionnaire-9 (PHQ-9) has demonstrated high sensitivity and specificity for depression across general, hospital, oncological, and hematological settings [13,14]. Its validity in Mexican urban and rural populations has been confirmed [15]. The Generalized Anxiety Disorder-7 scale (GAD-7) shows excellent internal consistency and a clear unidimensional structure for anxiety, with well-established clinical cutpoints [16,17]. Both instruments are brief enough for routine use in busy specialty clinics.

Despite this evidence base, systematic documentation of affective symptoms in Mexican HSCT patients - particularly at the moment of initial psychiatric evaluation - is absent from the literature. The most directly comparable published cohort is that of Ames et al. (2024), who applied PHQ-9 and GAD-7 prospectively in 205 patients evaluated prior to HSCT and found clinically meaningful rates of depression and anxiety at baseline [18]. No equivalent data exist for Mexican centers, where healthcare context, patient demographics, and referral patterns may differ substantially. That first encounter, before conditioning begins and while functional status is relatively preserved, may be the optimal window for psychosocial intervention: symptoms are detectable, the patient is accessible, and early support could influence the subsequent clinical course. We therefore conducted a retrospective, cross-sectional study to address this gap in Mexican HSCT cohorts. The primary objective was to describe the frequency and severity of depressive and anxiety symptoms, measured with the PHQ-9 and GAD-7, in patients entering the HSCT protocol at a major Mexican public tertiary center during their initial psychiatric evaluation. Secondary, exploratory objectives were to examine whether symptom levels differed by age group, sex, hematological diagnosis, and transplantation type, and to assess the correlation between PHQ-9 and GAD-7 scores. We did not formally prespecify these secondary analyses and report them as hypothesis-generating.

## Materials and methods

Study design and setting

This was a retrospective, cross-sectional, descriptive, observational study conducted at the Psychiatry Department of the Centro Medico Nacional "20 de Noviembre," ISSSTE, Mexico City - a university-affiliated tertiary referral center that performs approximately 60-80 BMT procedures annually. Institutional Review Board approval was obtained (RPI 144.2025, Comite de Etica e Investigacion, CMN "20 de Noviembre," ISSSTE); the study was classified as no-risk research under Article 17 of the Mexican Regulation of the General Health Law. Patient consent was not required given the retrospective, purely documentary design.

Participants

We reviewed clinical records of adult patients (age ≥18 years) who underwent initial psychiatric evaluation as part of the institutional BMT protocol between January 2024 and November 2025. Inclusion required documented PHQ-9 and GAD-7 scores from that first visit and a complete, accessible medical record. Patients were excluded if they were younger than 18, had not received both scales at the initial visit, had incomplete records, or were seen by psychiatry but not formally enrolled in the BMT protocol. All transplant candidates evaluated by the Psychiatry Department within the BMT protocol during the study period were screened, and every candidate meeting these criteria was consecutively included; no eligible patient was omitted, and the only exclusion was the single case with an incomplete GAD-7 record. Two investigators extracted data from the institutional clinical records using a standardized form that captured PHQ-9 and GAD-7 item and total scores, age, sex, hematological diagnosis, and planned transplantation type. A second reviewer checked each entry against the source record, and the supervising psychiatrist resolved discrepancies by consensus.

Instruments and variables

The PHQ-9 (9-item, range 0-27) [13] classifies depressive symptom severity as minimal (0-4), mild (5-9), moderate (10-14), moderately severe (15-19), and severe (20-27); a score ≥10 was used to define clinically significant depression, consistent with the original validation in general medical outpatients. We acknowledge that lower cutpoints (≥7 or ≥8) have shown superior diagnostic performance in oncology populations specifically (sensitivity 83-93%) [19,20], and that use of ≥10 may underestimate the true prevalence of depression in this sample; this is addressed as a limitation. The GAD-7 (7-item, range 0-21) [16] classifies anxiety as minimal (0-4), mild (5-9), moderate (10-14), and severe (15-21); a score ≥10 defined clinically significant anxiety. Both scales were administered in their Spanish-language versions validated for use in Mexican populations (PHQ-9 [15], GAD-7 [21]) by trained psychiatric residents during the standard clinical interview; this method may introduce social desirability bias, as patients may minimize symptoms when the interviewer is also their treating clinician.

Additional variables extracted from records included: age, sex, primary hematological diagnosis, and planned transplantation type (autologous or allogeneic).

Statistical analysis

We described quantitative variables with medians and interquartile ranges (IQR) given non-normal distributions confirmed by Shapiro-Wilk tests, or with means and standard deviations when appropriate. Categorical variables were summarized as frequencies and percentages. We reported proportions meeting cutpoint thresholds with exact (Clopper-Pearson) 95% confidence intervals (CIs). We estimated prevalence ratios between age groups as the ratio of subgroup proportions and derived their 95% CIs with the Katz log method; the corresponding p-values came from Fisher's exact tests. We defined age groups (18-35, 36-55, and ≥56 years) post hoc for exploratory comparison; they were not prespecified. Because documented PHQ-9 and GAD-7 scores at the initial visit were required for inclusion, we performed no imputation and analyzed complete cases only (N=103). We tested associations between age and categorical clinical variables with Kruskal-Wallis tests followed by post-hoc pairwise comparisons. Differences in the proportions of screen-positive patients across subgroups were evaluated with Fisher's exact tests or chi-square tests as appropriate. Spearman correlation was used to assess the relationship between PHQ-9 and GAD-7 total scores. All tests were two-tailed; alpha was set at 0.05. Analyses were performed with JMP 17 (SAS Institute Inc., Cary, NC) and Microsoft Excel 365 (Microsoft Corp., Redmond, WA).

## Results

Sample characteristics

**Figure 1:**
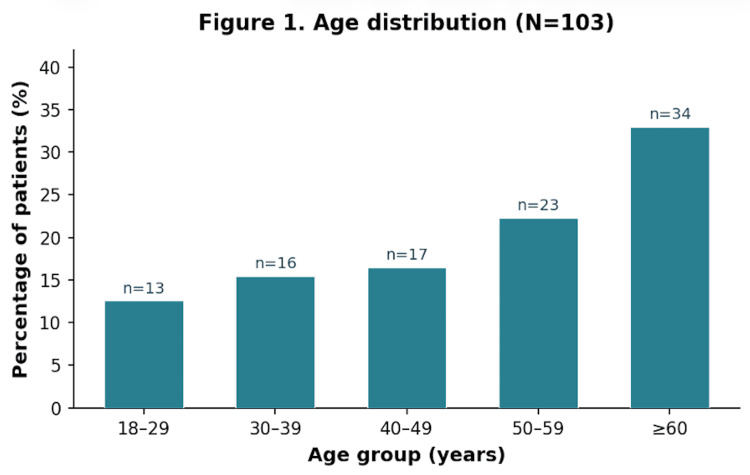
Age distribution of the study sample (N=103). Bars indicate percentage of patients per age group; counts (n) shown above each bar.

Of 104 records reviewed, 103 met the inclusion criteria and were analyzed. The single exclusion was due to an incomplete GAD-7 record. Median age was 51 years (IQR 38.5-60.5; range 19-75); mean was 48.5 years (standard deviation [SD] 14.5). Fifty-seven patients (55.3%) were men (Table 1). Figure 1 shows the age distribution; the largest proportion (33.0%) was aged 60 years or older.

**Table 1:** Sociodemographic and clinical characteristics (N=103)

Variable	n (%) or median (IQR)
Age, years — median (IQR)	51 (38.5–60.5)
Range	19–75
Sex — male	57 (55.3%)
Hematological diagnosis	
Multiple myeloma	50 (48.5%)
Acute lymphoblastic leukemia	17 (16.5%)
Non-Hodgkin lymphoma	11 (10.7%)
Hodgkin's lymphoma	8 (7.8%)
Acute myeloid leukemia	6 (5.8%)
Myelodysplastic syndrome	3 (2.9%)
Aplastic anemia	3 (2.9%)
Other hematological neoplasms	5 (4.9%)
Transplantation type	
Autologous	68 (66.0%)
Allogeneic	33 (32.0%)
Undefined in record	2 (1.9%)

**Figure 2:**
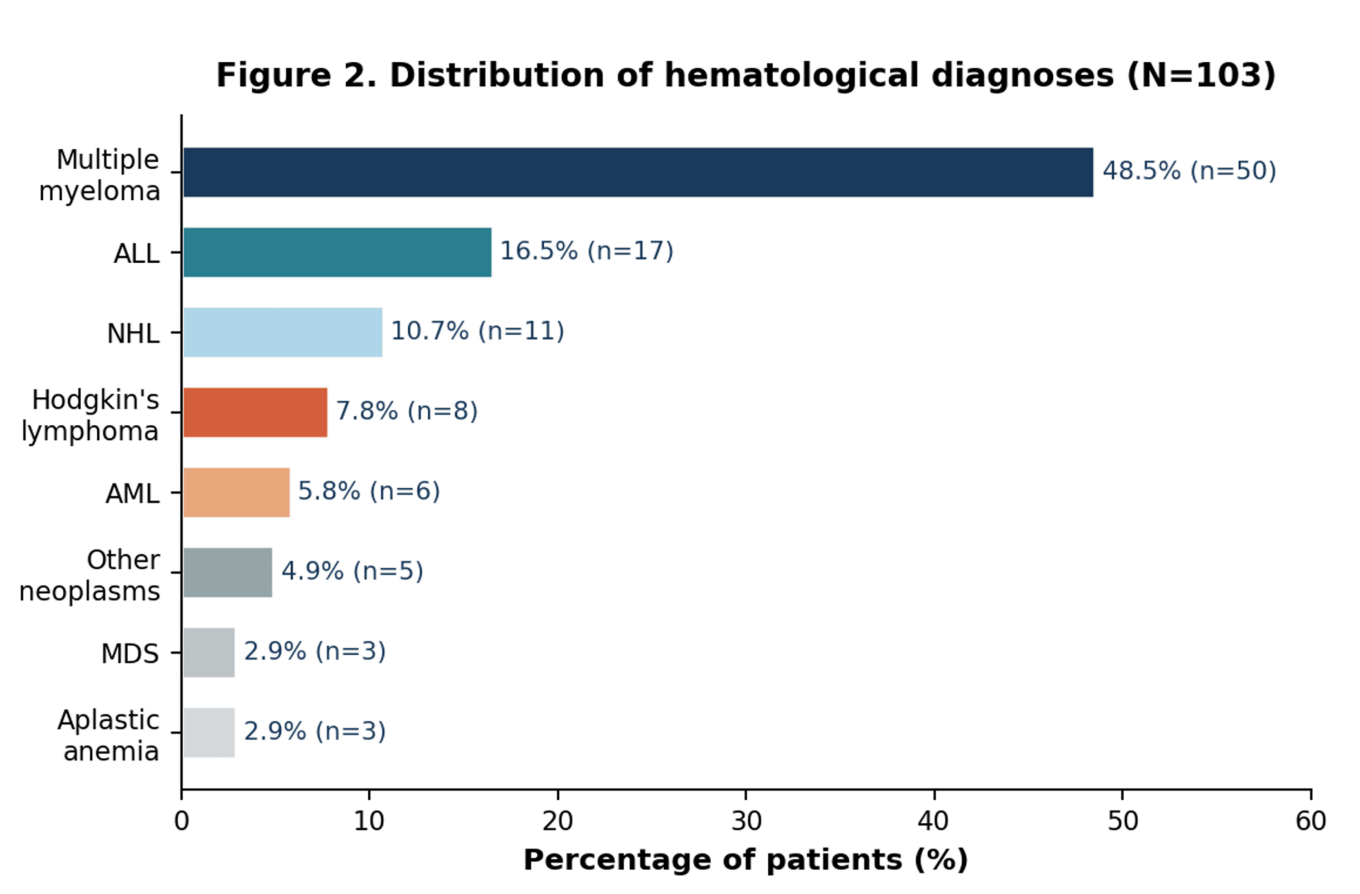
Distribution of hematological diagnoses. ALL: acute lymphoblastic leukemia; NHL: non-Hodgkin lymphoma; AML: acute myeloid leukemia; MDS: myelodysplastic syndrome.

Age differed significantly across hematological diagnoses (Kruskal-Wallis H = 42.68, df = 7; p<0.0001). Multiple myeloma patients were older (mean 57.2±9.5 years) and were predominantly scheduled for autologous transplantation, whereas patients with acute lymphoblastic leukemia were younger (mean 33.5±11.7 years) and were slated for allogeneic procedures. Autologous candidates were, on average, older than allogeneic candidates (53.5±12.0 vs. 37.4±13.1 years; Mann-Whitney U = 1835, z = 5.16; p<0.0001), a pattern consistent with the epidemiology of these diagnoses (Figure 2).

Depressive symptoms

**Figure 3:**
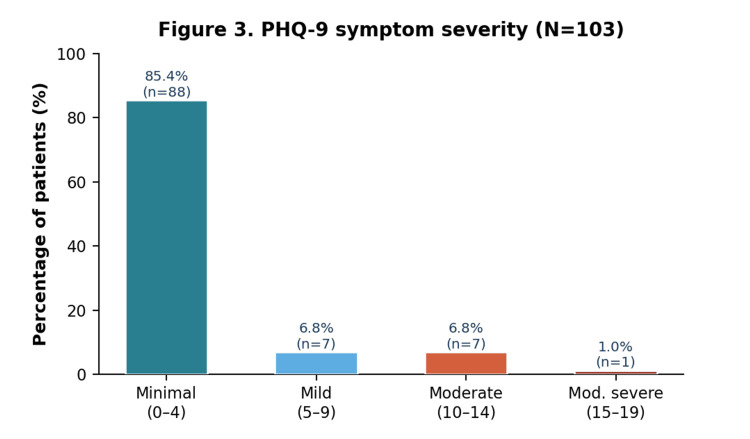
PHQ-9 symptom severity distribution. No patient reached the severe threshold (score 20–27). Error bars not applicable for frequency data.

The median PHQ-9 score was 0 (IQR 0-2; range 0-15). Eighty-eight patients (85.4%) scored in the minimal range, seven (6.8%) in the mild range, seven (6.8%) in the moderate range, and one (1.0%) in the moderately severe range; no patient reached the severe threshold. Using the PHQ-9 ≥10 cutpoint for clinically significant depression, eight patients met criteria (7.8%; 95% CI 4.0-14.6) (Table 2, Figure 3).

**Table 2:** Symptom severity distribution and prevalence of clinically significant cases

Category	n (%)	Median (IQR)	95% CI
PHQ-9 severity		0 (0–2)	—
Minimal (0–4)	88 (85.4%)		—
Mild (5–9)	7 (6.8%)		—
Moderate (10–14)	7 (6.8%)		—
Moderately severe (15–19)	1 (1.0%)		—
Severe (20–27)	0 (0%)		—
PHQ-9 ≥10 (depression)	8 (7.8%)	—	4.0–14.6
GAD-7 severity		0 (0–1.5)	—
Minimal (0–4)	89 (86.4%)		—
Mild (5–9)	12 (11.7%)		—
Moderate (10–14)	2 (1.9%)		—
Severe (15–21)	0 (0%)		—
GAD-7 ≥10 (anxiety)	2 (1.9%)	—	0.5–6.8
Co-occurrence (PHQ-9 ≥10 AND GAD-7 ≥10)	2 (1.9%)	—	0.5–6.8

Anxiety symptoms

**Figure 4:**
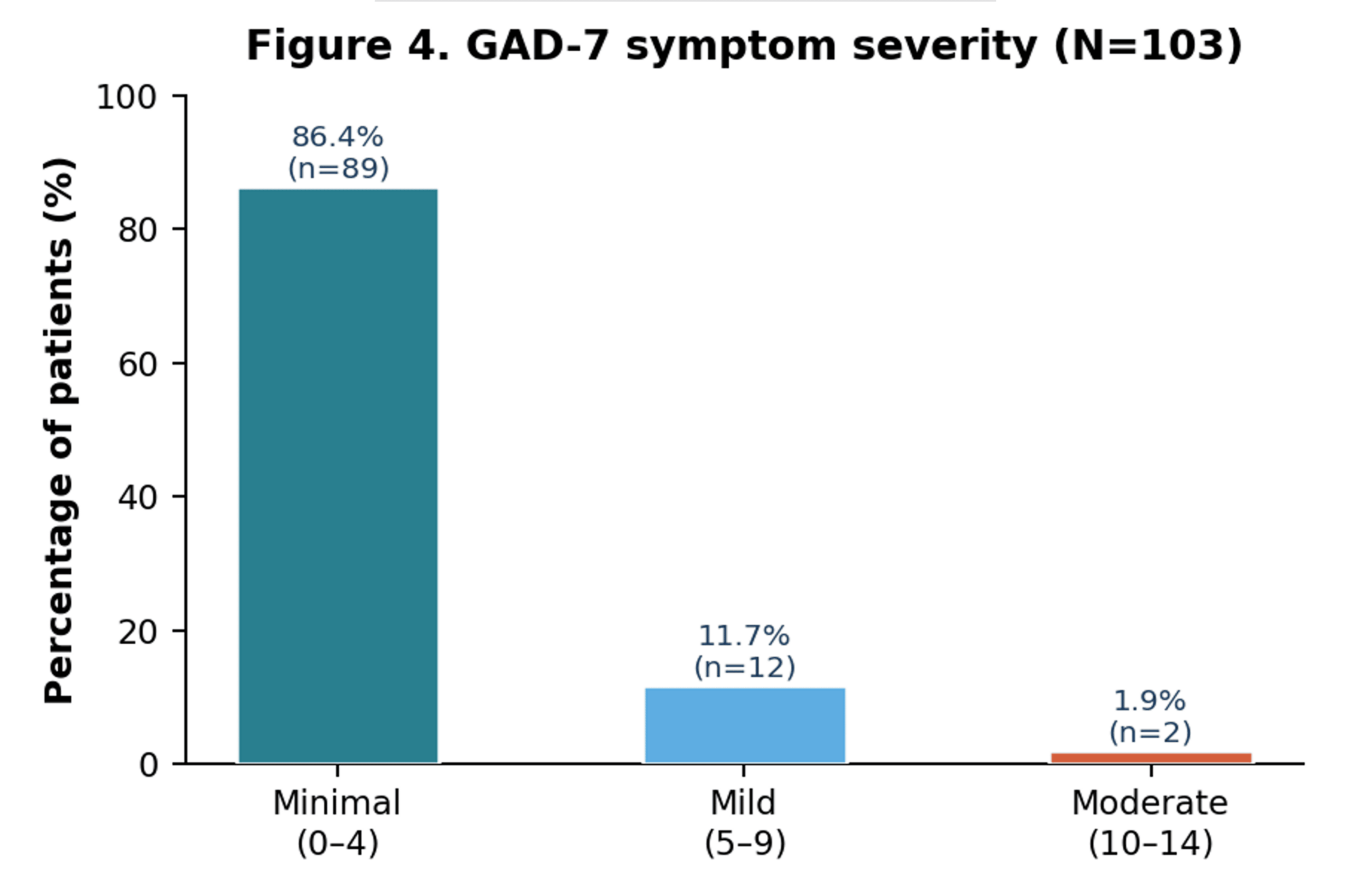
GAD-7 symptom severity distribution. No patient reached the severe threshold (score 15–21).

**Figure 5:**
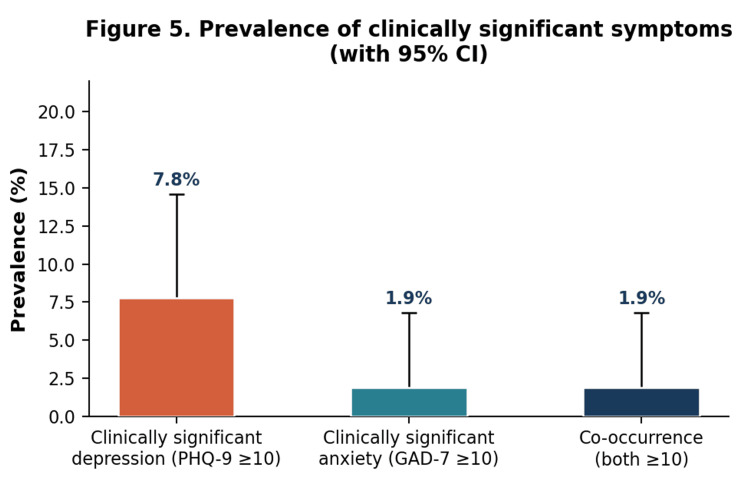
Prevalence of clinically significant depression (PHQ-9 ≥10), anxiety (GAD-7 ≥10), and co-occurrence, with 95% confidence intervals.

The median GAD-7 score was 0 (IQR 0-1.5; range 0-10). Eighty-nine patients (86.4%) scored in the minimal range, 12 (11.7%) in the mild range, and two (1.9%) in the moderate range; no patient reached the severe threshold. Using GAD-7 ≥10, two patients met criteria for clinically significant anxiety (1.9%; 95% CI 0.5-6.8). Both patients with clinically significant anxiety (GAD-7 ≥10) also met the threshold for clinically significant depression (PHQ-9 ≥10); accordingly, co-occurrence of both conditions was observed in the same two patients (1.9%; 95% CI 0.5-6.8) (Table 2, Figures 4-5).

Subgroup analyses

Depressive and anxiety symptom rates did not differ significantly by sex (all p>0.5 on Fisher's exact tests) or by transplantation type (autologous vs. allogeneic; all p>0.5). No statistically significant association was found between specific hematological diagnoses and PHQ-9 or GAD-7 continuous scores (Kruskal-Wallis, all p>0.1 after removing the age-driven confounding).

An exploratory analysis of mild anxiety (GAD-7 5-9) by age group showed that patients aged 18-35 years had this symptom level approximately four times more often than older groups. The prevalence of mild anxiety in the 18-35-year group was 30.0% vs. 7.1% in the 36-55-year group (prevalence ratio 4.2; 95% CI 1.17-15.10; Fisher exact p=0.026). This finding should be interpreted cautiously: the extremely wide confidence interval (spanning more than 13 units) indicates substantial estimation instability, likely reflecting the small number of events. A similar directional pattern was observed when the youngest group was compared with patients aged 56 years and older.

PHQ-9 / GAD-7 correlation

**Figure 6:**
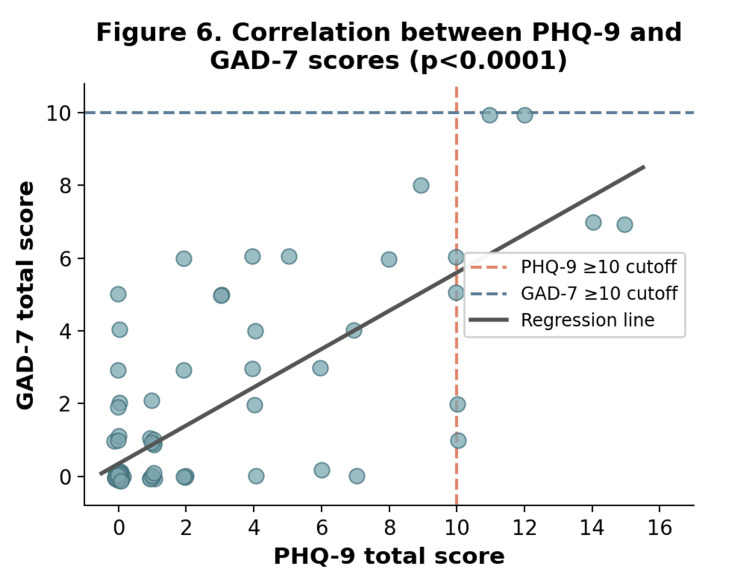
Scatter plot of PHQ-9 vs. GAD-7 scores (N=103). Dashed lines indicate clinical cutpoints (score ≥10). Regression line shown in black (Spearman rho=0.66, p<0.0001). Jitter added for visibility.

**Figure 7:**
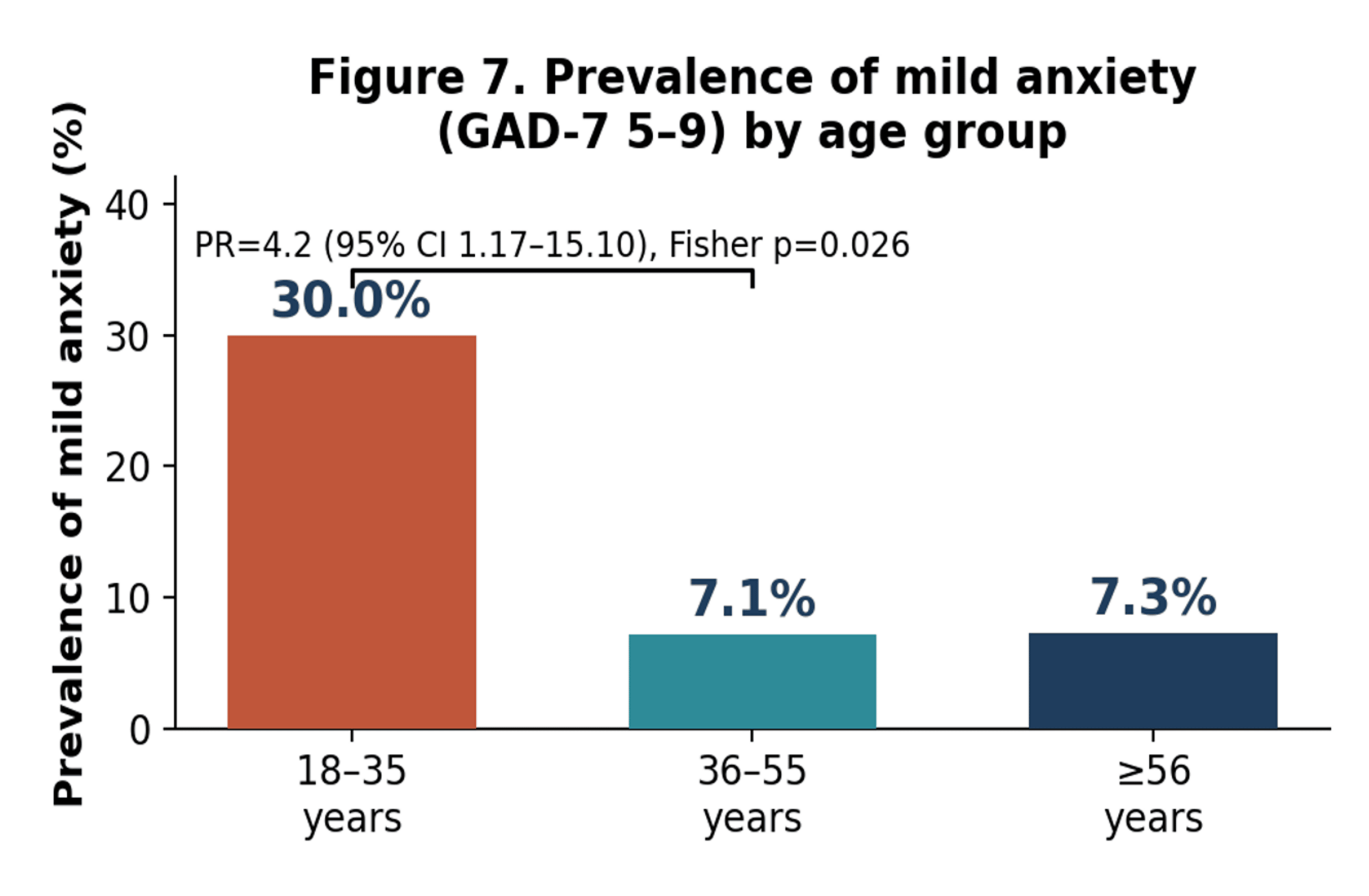
Prevalence of mild anxiety (GAD-7 5–9) by age group. The 18–35-year group showed a fourfold higher prevalence compared with the 36–55-year group (PR=4.2; 95% CI 1.17–15.10; p=0.026).

A significant positive correlation was found between PHQ-9 and GAD-7 total scores (Spearman rho=0.66; p<0.0001), indicating that patients with higher depressive symptom burden also tended to report greater anxiety, consistent with the well-described comorbidity between both domains in medically ill populations (Figures 6-7).

## Discussion

We found that clinically significant depression occurred in 7.8% of patients and clinically significant anxiety in 1.9% at the time of their first psychiatric consultation within the HSCT protocol - well below the 20-50% and 20-33% figures, respectively, commonly cited in the international literature [4-7,22-24]. The most directly comparable published cohort used the same instruments in 205 pre-HSCT patients; Ames et al. (2024) reported higher rates of baseline depression and anxiety, a discrepancy consistent with the methodological and contextual differences detailed below [18]. Rather than treating the gap as a puzzling discrepancy, three contextual factors largely account for it.

First, timing matters. Most prior studies assessed patients during conditioning, hospitalization, or early post-transplant follow-up, when physical toxicity, social isolation, and prognostic uncertainty are at their peak [2,3,25,26]. Our cohort was captured at the opposite end of that arc: patients had just been deemed eligible for HSCT and were still living in the community, often with intact support networks and preserved daily functioning. Affective symptoms at this pre-conditioning baseline would be expected to be lower than at any subsequent phase of treatment. Notably, Kuba et al. found in a prospective population-based German cohort that pre-HSCT depression was actually lower than in the general population (relative risk [RR] 0.56; 95% CI 0.39-0.81), while pre-HSCT anxiety was modestly elevated (RR 1.31; 95% CI 1.02-1.68) [6]. The very low anxiety rates in our sample (1.9% clinically significant vs. up to 29% in Kuba et al. at pre-transplant assessment) may therefore reflect genuine between-sample differences in referral patterns, patient selection, and cultural expression of anxiety symptoms, rather than a floor effect alone.

Second, selection bias runs in our direction. Patients who reach formal BMT candidacy have, by definition, passed through a clinical sieve: those with severe psychiatric comorbidity, poor adherence histories, or compromised functional status are often deferred or excluded earlier in the hematological workup [11,12,27]. Our sample therefore skews toward psychosocially stable individuals. This phenomenon - reported in liver, lung, and cardiac transplant programs as well - systematically depresses prevalence estimates at point-of-referral evaluations.

Third, validated screeners like the PHQ-9 and GAD-7 capture specific symptom clusters reliably but may not register subtler forms of distress - existential dread, anticipatory grief, illness-related identity disruption - that do not translate into high numerical scores yet still affect quality of life and adherence [28]. Our near-zero medians should not be read as evidence of emotional equanimity; they reflect the psychometric specificity of these instruments at a moment when overt syndromal symptoms have not yet consolidated.

Despite the modest overall rates, two findings carry direct clinical weight. The 7.8% prevalence of clinically significant depression in a population that has often been pre-selected for stability is not negligible: in a program that evaluates 60-80 patients annually, this translates to roughly five or six patients per year who enter the transplant pipeline with a treatable depressive disorder. Without systematic screening, these cases go undetected until symptoms worsen under the stress of conditioning, potentially impairing immunological recovery and clinical outcomes [9,10].

Younger patients (18-35 years) also stood out: they were four times more likely to present mild anxiety than those in the 36-55-year bracket. This finding fits a broader pattern documented in psycho-oncology: younger adults facing serious hematological diagnoses frequently experience a collision between the illness and developmental goals - career interruption, fertility concerns, altered relationship trajectories - that generates psychological distress qualitatively different from, and often more intense than, the reactions seen in older patients who have already consolidated those life structures [28-30]. This interpretation requires an important qualification, however: in this sample, younger patients (18-35 years) are almost exclusively patients with acute lymphoblastic leukemia scheduled for allogeneic transplantation, while older patients are predominantly multiple myeloma patients scheduled for autologous procedures. The effect attributed to age is therefore thoroughly confounded with hematological diagnosis and transplantation type - two variables that independently predict psychological distress and carry very different prognoses, treatment intensities, and social disruptions. The present data cannot disentangle these effects, and this confounding should be acknowledged when interpreting the age-anxiety association. Even subthreshold anxiety may affect procedural adherence and quality of life in younger patients, and brief targeted interventions (psychoeducation, acceptance-based approaches, short-term cognitive-behavioral techniques) delivered proactively could be cost-effective.

The strong PHQ-9/GAD-7 correlation (rho=0.66) replicates the well-established comorbidity between depressive and anxiety spectra in medically ill populations and supports an integrated assessment approach: screening for one dimension should prompt evaluation of the other, and pharmacological or psychotherapeutic interventions should address both domains simultaneously when present [7,22,23].

Limitations

This study has several limitations that bear on the interpretation of its results. The retrospective design precluded standardized collection of variables such as prior psychiatric history, psychotropic medication use, perceived social support, and coping styles - all of which predict affective outcomes in HSCT populations [4,5,9-12]. The cross-sectional, single-timepoint design cannot characterize symptom trajectories or establish causal pathways. We did not calculate an a priori sample size, because the study drew on a fixed, consecutively enrolled clinical cohort. The small number of screen-positive patients (n=8 for depression; n=2 for anxiety) left subgroup analyses underpowered, so the absence of significant associations by sex or transplantation type is inconclusive rather than evidence of equivalence. The age-anxiety finding (Fisher's exact p=0.026; PR 4.2, 95% CI 1.17-15.10) carries a wide confidence interval reflecting estimation instability and should be considered exploratory pending replication. The convenience sampling and single-center setting limit generalizability, as institutional practices, patient demographics, and referral patterns differ across centers and health systems. The PHQ-9 ≥10 cutpoint, while standard in general medical populations, may underestimate the true prevalence of depression in oncological samples: studies in cancer patients have found that lower thresholds (PHQ-9 ≥7 or ≥8) optimize sensitivity and specificity in this population [19,20]. Marked floor effects - median scores of 0 for both PHQ-9 and GAD-7 - limit the variability available for correlation and subgroup analyses; accordingly, the Spearman rho of 0.66 should be interpreted with caution in the context of this restricted range. Administration of scales by psychiatric residents during the clinical interview introduces a potential social desirability bias, as patients may systematically underreport symptoms to the clinician who will also make decisions about their transplant eligibility. Results for PHQ-9 item 9 (suicidal ideation), which carries particular clinical relevance given that suicide rates in cancer patients are two to three times higher than in the general population, were not reported as a separate outcome. Finally, although both scales are validated in Mexican populations [15,21], cultural variation in symptom expression and response styles could influence scores in this specific clinical context.

## Conclusions

In a cohort of 103 patients evaluated psychiatrically at entry into an HSCT protocol, clinically significant depression was present in 7.8% and clinically significant anxiety in 1.9%. These rates are lower than those reported in studies conducted during more acute phases of the transplantation process, reflecting the timing of the evaluation and the pre-selection of medically stable candidates. Younger patients concentrated a disproportionate share of mild anxiety, though this effect is confounded with hematological diagnosis and transplantation type and should be interpreted cautiously. The strong correlation between PHQ-9 and GAD-7 scores reinforces the rationale for integrated affective screening. These findings support the routine incorporation of validated brief scales at the initial psychiatric encounter and underscore the need for longitudinal cohort studies that track affective symptoms from pre-transplant evaluation through post-transplant recovery.
